# Ruminal Microbes Exhibit a Robust Circadian Rhythm and Are Sensitive to Melatonin

**DOI:** 10.3389/fnut.2021.760578

**Published:** 2021-10-25

**Authors:** Jialiang Ouyang, Mengzhi Wang, Dengpan Bu, Lu Ma, Fuyuan Liu, Chun Xue, Chao Du, Ahmad Aboragah, Juan J. Loor

**Affiliations:** ^1^State Key Laboratory of Sheep Genetic Improvement and Healthy Production, Xinjiang Academy of Agricultural and Reclamation Sciences, Shihezi, China; ^2^Institute of Animal Science, State Key Laboratory of Animal Nutrition, Chinese Academy of Agricultural Sciences (CAAS), Beijing, China; ^3^College of Animal Science and Technology, Yangzhou University, Yangzhou, China; ^4^Chinese Academy of Agricultural Sciences-World Agroforestry Centre (CAAS-ICRAF) Joint Lab on Agroforestry and Sustainable Animal Husbandry, World Agroforestry Center, East and Central Asia, Beijing, China; ^5^Department of Animal Sciences and Division of Nutritional Sciences, University of Illinois, Urbana, IL, United States

**Keywords:** lactating cows, rumen, melatonin, flora structure, circadian rhythm

## Abstract

Gut hormones are not only able to regulate digestive, absorptive, and immune mechanisms of the intestine through biological rhythms, but impact the host through their interactions with intestinal microorganisms. Whether hormones in ruminal fluid have an association with the ruminal ecology is unknown. Objectives of the study were to examine relationships between the diurnal change in ruminal hormones and microbiota in lactating cows, and their associations *in vivo* and *in vitro*. For the *in vivo* study, six cows of similar weight (566.8 ± 19.6 kg), parity (3.0 ± 0.0), and milk performance (8,398.7 ± 1,392.9 kg/y) were used. They were adapted to natural light for 2 weeks before sampling and fed twice daily at 07:00 a.m. and 14:00 p.m. Serum, saliva, and ruminal fluid samples were collected at 02:00, 10:00, and 18:00 on the first day and 06:00, 14:00, and 22:00 on the second day of the experimental period. The concentrations of melatonin (MLT), growth hormone (GH), and prolactin (PRL) were measured *via* radioimmunoassay, whereas amplicon sequencing data were used to analyze relative abundance of microbiota in ruminal fluid. JTK_CYCLE analysis was performed to analyze circadian rhythms of hormone concentrations as well as the relative abundance of microbiota. For the *in vitro* study, exogenous MLT (9 ng) was added into ruminal fluid incubations to investigate the impacts of MLT on ruminal microbiota. The results not only showed that rumen fluid contains MLT, but the diurnal variation of MLT and the relative abundance of 9% of total rumen bacterial operational taxonomic units (OTUs) follow a circadian rhythm. Although GH and PRL were also detected in ruminal fluid, there was no obvious circadian rhythm in their concentrations. Ruminal MLT was closely associated with Muribaculaceae, Succinivibrionaceae, Veillonellaceae, and Prevotellaceae families *in vivo*. *In vitro*, these families were significantly influenced by melatonin treatment, as melatonin treatment increased the relative abundance of families Prevotellaceae, Muribaculaceae while it reduced the relative abundance of Succinivibrionaceae, Veillonellaceae. Collectively, ruminal microbes appear to maintain a circadian rhythm that is associated with the profiles of melatonin. As such, data suggest that secretion of melatonin into the rumen could play a role in host-microbe interactions in ruminants.

## Introduction

Intestinal microbial research has achieved great success in humans and mice. A host of studies demonstrated that gut microbes play an important role in regulating digestion, absorption and metabolism of the host, and these microbes are also able to induce or prevent inflammation of the digestive tract (1–3). Intestinal microorganisms can modulate lipid, sugar and protein metabolism in the host, hence, are closely associated with a variety of diseases such as type 2 diabetes (T_2_D) (4), Parkinson's disease (5), colon cancer (6, 7) and even human immunodeficiency virus (HIV) infection (8).

Gut microbes exhibit robust circadian rhythms, and their circadian patterns of relative abundance, absolute abundance and metabolomics were shown to be affected by the biological rhythm of the host (9, 10), which are largely regulated by circadian secretion of several hormones including Melatonin (MLT) (11, 12).

The intestinal tract, in particular, is capable of hormonal secretion according to a circadian rhythm, and some of these hormones are able to influence the intestinal bacteria. For instance, some of these hormones could induce locally resident microbes to release functional cytokines or chemokines (13). In turn, the microbiota is able to stimulate intestinal endocrine cells to release gut peptides and hormones, effectively resulting in interactions with the host circadian clock (14). Clearly, a growing body of literature is highlighting the existence of crosstalk between host and intestinal microbiota partly due to gut hormones.

The MLT is one of the most-essential hormones involved in regulating circadian rhythms in the host, and the complex effects of this hormone on the gut microbiome has just begun to be studied (15). For instance, Ma et al. (16) demonstrated a firm connection among MLT, gut microbiota and mucosal immune cells. Paulose et al. (17) and Paulose and Cassone (18) also reported that MLT modulated in a circadian fashion intestinal microorganisms such as *Enterobacter aerogenes* in humans. In addition, Zhu et al. (19) reported that MLT treatment could reduce the relative abundance of Bacteroidetes from 58.93 to 41.63% while increasing Firmicutes in the intestine of mice with colitis.

Although host-microbe communication is an integral component of normal physiological mechanisms in the human body, specific factors that coordinate this communication in ruminants remain to be fully established. Microbial density in ruminal fluid can be as high as 10^9^ cells/mL (20), and a number of studies have associated specific microbiota profiles with phenotypes such as milk yield in dairy cows (21–23). For example, Mu et al. (24) reported that abundance of *Prevotella* spp. in ruminal fluid was negatively correlated with milk production, whereas abundance of Succinivibrionaceae family was positively associated with milk yield (25). Furthermore, hormones such as growth hormone (GH), prolactin (PRL) and MLT in ruminants are closely linked with lactation performance (26, 27). We hypothesized that, similar to humans, hormones and ruminal microbes in ruminants follow a circadian rhythm, and the rhythmic hormones can modulate the profiles of specific bacteria. To address this hypothesis, we investigated the diurnal variation of GH, PRL, and MLT concentrations along with ruminal microbiota profiles. The linkages between these hormones and bacteria were explored *in vivo* and *in vitro*.

## Results

### Diurnal Variation of MLT, GH, and PRL in Serum, Saliva, and Ruminal Fluid

**Figure 1:**
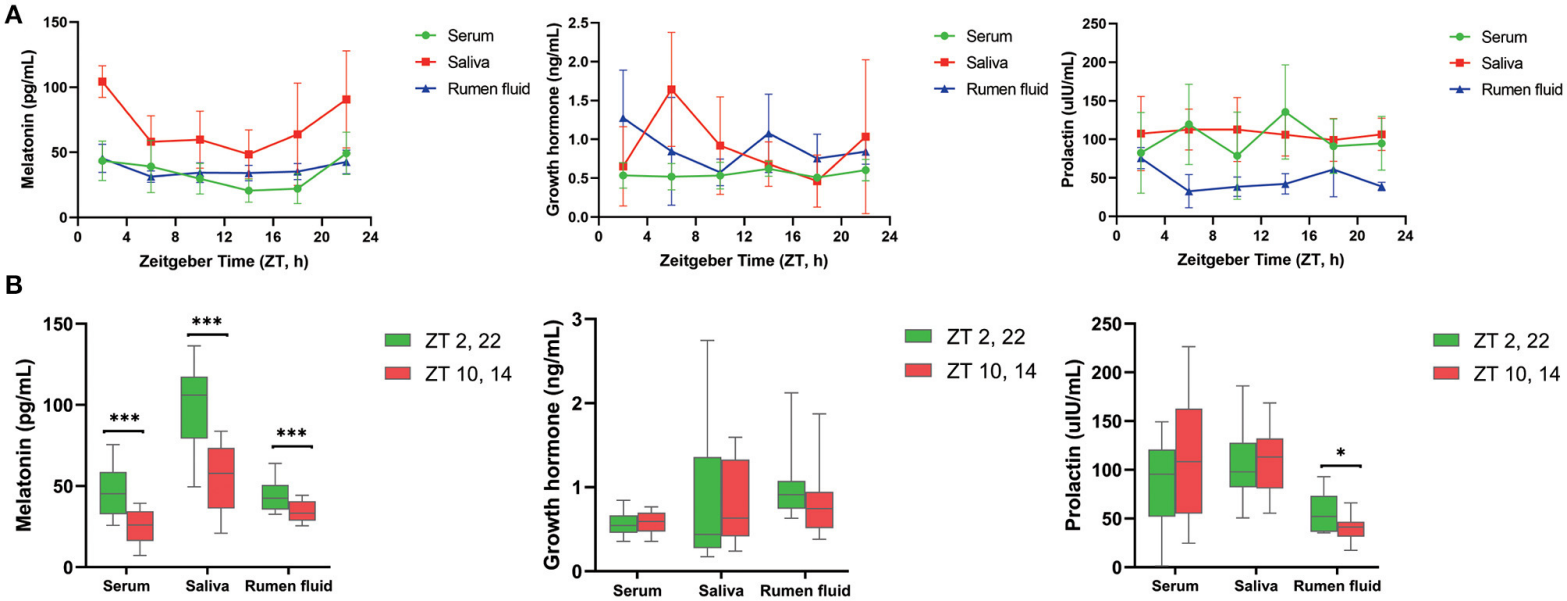
Diurnal change of concentrations of three hormones in different samples. **(A)** Line graphs show the diurnal concentration of hormones comprising melatonin (left), growth hormone (middle) and prolactin (right) in serum, saliva and rumen fluid. **(B)** Boxplot figures show the difference of three hormones concentration between dark (ZT2, 22) and light conditions (ZT10, 14) in serum, saliva and rumen fluid of dairy cows. Conditions are color coded (see legend). ZT, Zeitgeber time. *P < 0.05, **P < 0.01, and ***P < 0.001.

As shown in Table 1, MLT was detectable in serum, saliva as well as ruminal fluid, and JTK_CYCLE analysis indicated that concentrations followed a circadian rhythm (Benjamini–Hochberg *q*-value, BH.Q < 0.05, Bonferroni-adjusted *p*-value, ADJ. *P* < 0.05). The trend of diurnal variation of MLT concentrations in these three biological fluids was similar, with higher concentrations at 2:00 and 22:00 compared with 10:00 and 14:00 (*P* < 0.001, Figure 1A). The concentrations of GH and PRL (Figures 1A,B) in serum, saliva, and ruminal fluid did not follow an obvious circadian oscillation (BH. *Q* > 0.05, ADJ. *P* > 0.05), despite the fact that the concentration of PRL in rumen fluid at 2:00 and 22:00 was significantly higher than that at 10:00 and 14:00.

**Table 1:** Melatonin (MLT) concentration in serum, saliva and rumen fluid (pg/mL).

**Item**	**Time**						**SEM**	***P*** **-value**
	**02:00**	**06:00**	**10:00**	**14:00**	**18:00**	**22:00**		
Serum	43.62^ab^	39.13^abc^	29.75^bcd^	20.63^d^	22.23^cd^	49.32^a^	5.895	<0.001
Saliva	104.37^a^	58.13^bc^	59.80^bc^	48.56^c^	63.95^bc^	90.72^ab^	10.948	0.002
Rumen fluid	45.49^a^	31.38^c^	34.45^bc^	34.14^bc^	35.36^bc^	42.64^ab^	3.119	0.002

*In the same row, values with no letter or the same letter superscripts mean no significant difference (P > 0.05), whereas with different superscripts mean significant difference (P < 0.05)*.

### Circadian Rhythm in Ruminal Microbiota Profiles

**Figure 2:**
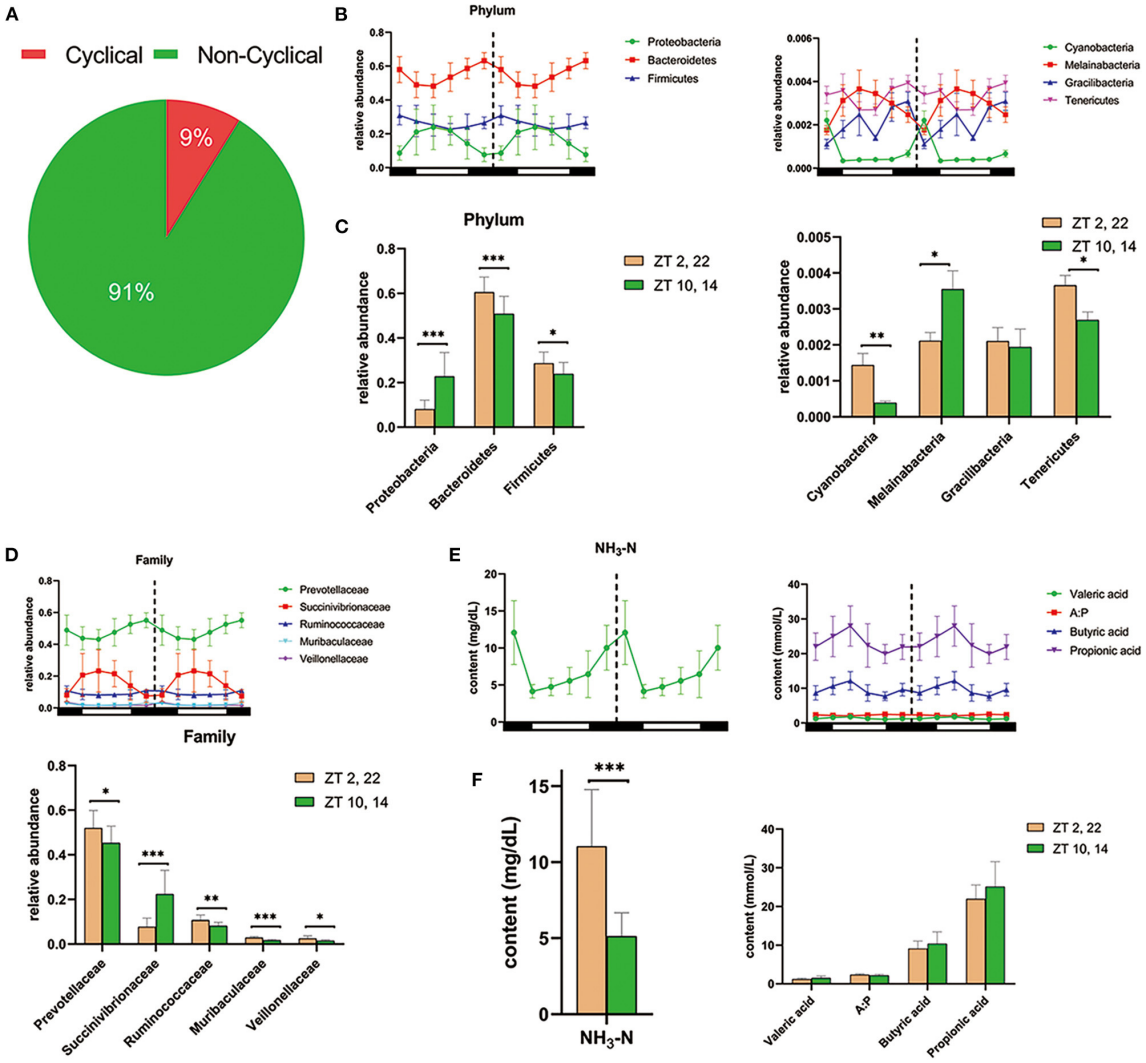
Diurnal rhythms of ruminal microbiota and fermentation parameters in dairy cows. **(A)** Pie chart shows the percentages of cyclical OTUs and non-cyclical OTUs based on the results of JTK_CYCLE analysis. **(B)** The double-plot line graph—where the second cycle is a duplicate of the first cycle following the dashed line—shows the diurnal change in relative abundance of the three most predominant phylum (left) and four other phyla (right) at each time point (*n* = 6 per time point). All of these phyla were cycling based on the JTK_CYCLE analysis (that is ADJ. *P* < 0.05 and BH. *Q* < 0.05). Black and white boxes indicate day and night, respectively. Different colored lines represent different phylum (see legend) **(C)** Bar graphs show the average relative abundance of the seven phyla depicted in **(B)** during dark (ZT2, 22) and light conditions (ZT10, 14). Conditions are color coded (see legend). *P < 0.05, **P < 0.01, and ***P < 0.001. **(D)** Upper double-plot line graph shows the diurnal relative abundance of five predominant family, which were cyclical according to the JTK_CYCLE analysis. Lower bar graph shows the mean relative abundance of the five predominant circadian family under the dark condition and light condition. **(E)** Colored line graphs present the content of dominant circadian rumen fermentation parameters in JTK_CYCLE analysis at each time point (*n* = 6 per time point). NH_3_-N, ammonia nitrogen; A:P, the ratio of acetate to propionate. **(F)** The average content of rhythmical rumen fermentation parameters under the dark condition (yellow) and light condition (green). *P < 0.05, **P < 0.01, and ***P < 0.001.

*In vivo*, amplicon sequencing generated a total of 3,156,355 high-quality sequences and an average of 87,677 ± 2,506 sequences per sample, which were assigned to 2,965 OTUs. Furthermore, JTK_CYCLE analysis identified 262 OTUs exhibited a significant circadian rhythm (BH. *Q* < 0.05, ADJ. *P* < 0.05) and underscored that ~9% of ruminal microbes followed an obvious circadian rhythm (Figure 2A). At the phylum level, JTK_CYCLE analysis revealed that three predominant ruminal species, Proteobacteria, Bacteroidetes, and Firmicutes followed a circadian pattern, with relative abundance of Bacteroidetes and Firmicutes being higher in dark (Zeitgeber time, ZT 2,22) compared with light conditions (ZT 10,14; *P* < 0.05; Figures 2B,C). In addition, the phyla Cyanobacteria, Melainabacteria, Gracilibacteria, and Tenericutes also followed a circadian rhythm (BH. *Q* < 0.05, ADJ. *P* < 0.05). Except for Gracilibacteria, relative abundance of these microorganisms was clearly influenced by the light/dark cycle (Figures 2B,C). At the family level, JTK_CYCLE analysis revealed that Prevotellaceae, Succinivibrionaceae, Ruminococcaceae, Muribaculaceae, and Veillonellaceae followed a clear circadian oscillation (BH. *Q* < 0.05, ADJ. *P* < 0.05). Furthermore, apart from Succinibrionaceae that exhibited markedly higher abundance during light conditions (*P* < 0.001), the other four bacterial families substantially increased in dark conditions (*P* < 0.05; Figure 2D).

### Circadian Rhythms in Ruminal Fermentation Parameters

As shown in Figure 2E, there was a clear circadian rhythm (BH. *Q* < 0.05, ADJ. *P* < 0.05) for ruminal ammonia nitrogen (NH_3_-N), valerate, butyrate, propionate as well as the ratio of acetate to propionate (A:P). Concentrations of NH_3_-N in ruminal fluid during light conditions were markedly lower than that during dark conditions (*P* < 0.001) (Figure 2F). Furthermore, the content of propionate, butyrate, and valerate was numerically higher during light conditions (*P* > 0.05; Figure 2F).

### Linkages Between Ruminal Microbes and Melatonin *in vivo*

**Figure 3:**
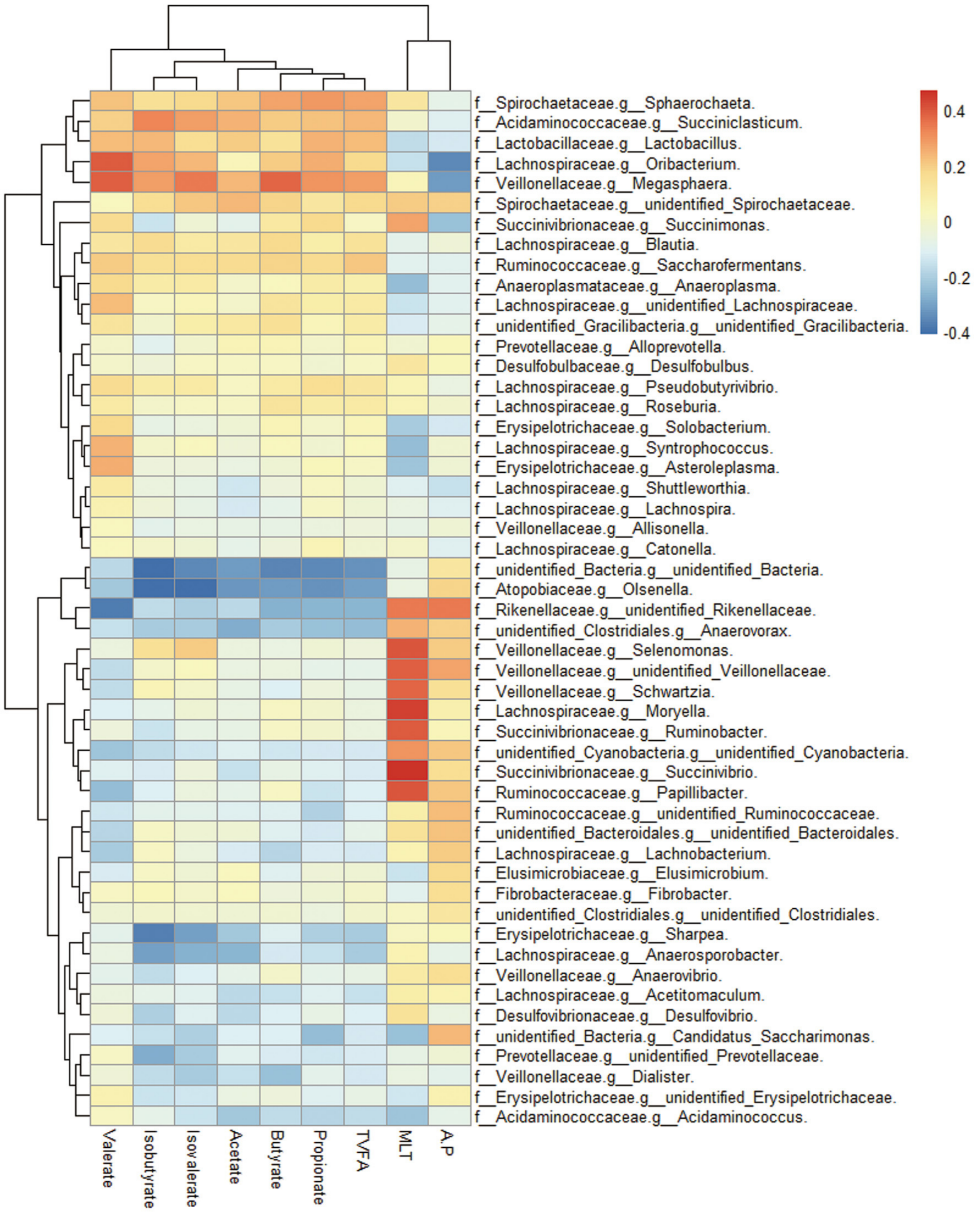
The ruminal microbes correlated with ruminal melatonin and fermentation parameters. Cluster heatmap figure shows the correlation between bacterial family in the rumen and several ruminal metabolites compassing melatonin and volatile fatty acids. MLT, melatonin; TVFA, total volatile fatty acids; A:P, the ratio of acetate to propionate; f, family; g, genus.

Correlations between ruminal bacterial families and ruminal melatonin or volatile fatty acids are reported in Figure 3. Families Muribaculaceae, Rikenellaceae, unidentified Cyanobacteria, Defluviitaleaceae, Veillonellaceae, Spirochaetaceae, and Protevotellaceae were positively correlated with ruminal melatonin. In contrast, unidentified Bacteria, Anaeroplasmataceae, and Rhodobacteraceae were negatively correlated with ruminal melatonin (Figure 3); genus *Succinivibrio, Ruminobacter, Selenomonas*, and *Moryella* also had a markedly negative correlation with ruminal melatonin (Supplementary Figure S1). In addition, in contrast to Rhodobacteraceae, the families Muribaculaceae, Rikenellaceae, unidentified Cyanobacteria and Protevotellaceae were negatively associated with propionate (Figure 3).

### Effect of MLT on Prevotellaceae and Succinivibrionaceae *in vitro*

The *in vitro* simulated fermentation experiment was conducted to investigate the impacts of melatonin on ruminal microbiota *ex vivo*. A total of three different treatments were included: (i) non-rumen fluid + non-melatonin (CK0); (ii) rumen fluid + non-melatonin (CK1); (iii) rumen fluid + melatonin (MLT). The result showed that melatonin concentration was influenced by both of time and treatment *in vitro* and, compared with CK0, the decrease rate of melatonin in the CK1 and MLT groups was significantly greater (Table 2). As for bacteria, amplicon sequencing of the partial 16S rRNA gene generated a total of 2,532,697 high-quality sequences and an average of 60,302 ± 1,856 sequences per sample, which were assigned to 1,639 OTUs. However, the average distinct OTUs count in CK1 was 1,158 ± 42, which was significantly different from MLT (1,226 ± 52; *P* = 0.001).

**Table 2:** Melatonin concentration of *in vitro* fermentation (pg/mL).

**Item**	**Treatment**			**SEM**	**Time**	**Treatment**	**Ti × Tr**
	**CK0**	**CK1**	**MLT**				
0 h	48.17^b^	50.39^b^	99.83^a^		^***^	^***^	^**^
4 h	47.90^b^	46.48^b^	84.64^a^				
8 h	34.19^b^	41.82^b^	82.38^a^				
12 h	46.40^b^	42.58^b^	87.01^a^	12.899			
16 h	29.54^b^	28.72^b^	79.42^a^				
20 h	31.88^b^	24.91^b^	52.96^a^				
24 h	28.08^ab^	15.79^b^	57.82^a^				
Decrease rate (pg·mL^−1^·h^−1^)	0.84^b^	1.44^a^	1.75^a^				

*In the same row, values with no letter or the same letter superscripts mean no significant difference (P > 0.05), whereas with different superscripts mean significant difference (P < 0.05). CK0, non-rumen fluid + non-melatonin; CK1, rumen fluid + non-melatonin; MLT, rumen fluid + melatonin*.

** *P < 0.01, and*

*** *P < 0.001*.

**Figure 4:**
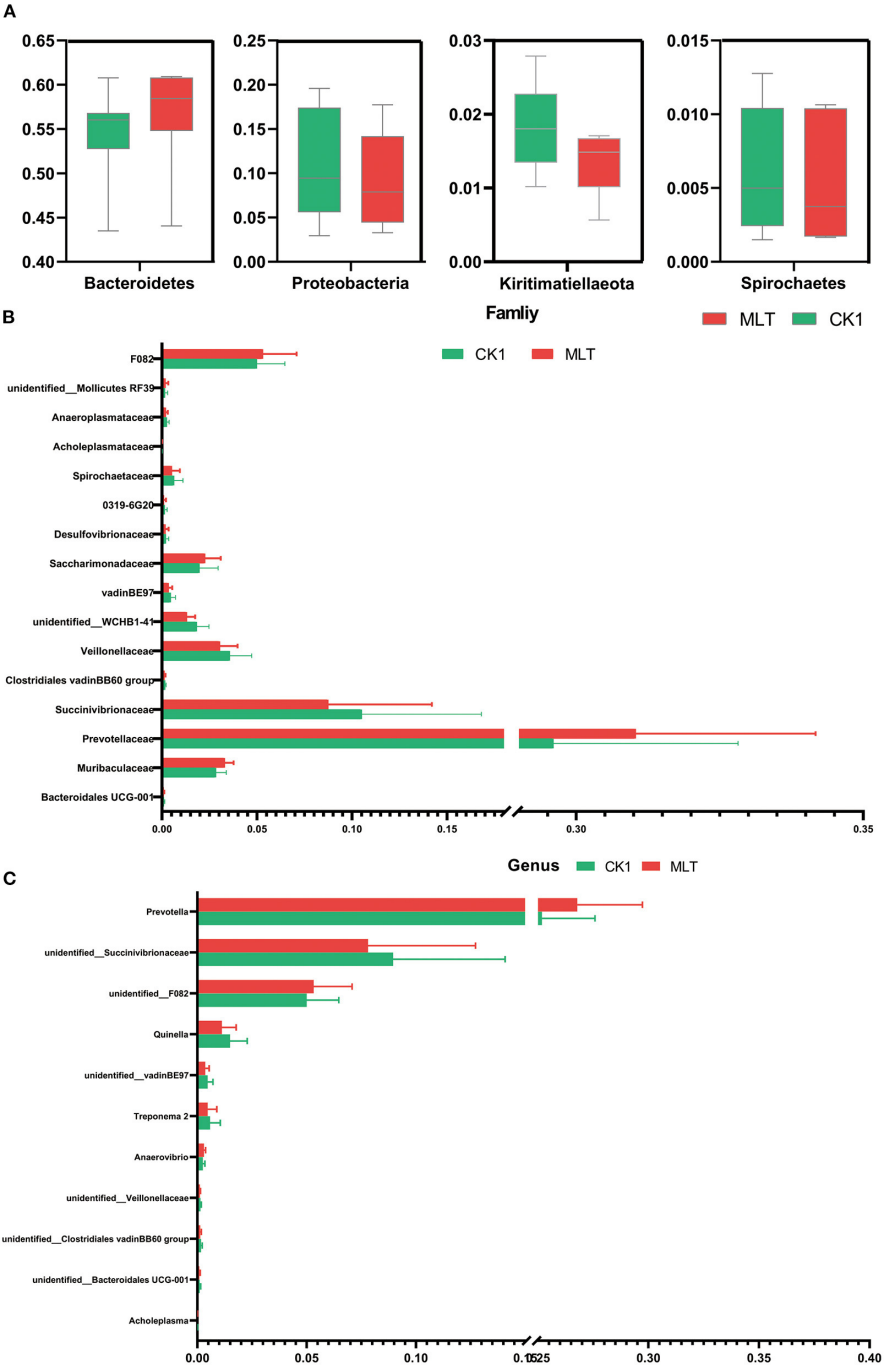
Melatonin impacted the ruminal microbiota *in vitro* fermentation. **(A)** Boxplot graphs show the average relative abundance of bacterial phyla that were significantly different (*P* < 0.05) based on paired *T*-tests between CK1 (green box) and MLT (red box) treatments. **(B)** Bar graph shows the average relative abundance of bacterial family that were significantly different (*P* < 0.05) based on the paired *T*-tests between CK1 and MLT treatments. **(C)** Bar graph shows the average relative abundance of bacterial genus that were significantly different (*P* < 0.05) based on the paired *T*-tests between CK1 and MLT treatments. CK0, non-rumen fluid + non-melatonin; CK1, rumen fluid + non-melatonin; MLT, rumen fluid + melatonin.

Although the Shannon index and Beta diversity *in vitro* were not altered by melatonin treatment (Supplementary Figure S2), this hormone increased the relative abundance of phyla Bacteridetes and decreased Proteobacteria, Kiritimatiellaeota and Spirochaetes (*P* < 0.05; Figure 4A). At the family level, melatonin treatment increased the relative abundance of Prevotellaceae, F082, Muribaculaceae, and Saccharimonadaceae; however, it reduced the relative abundance of Succinivibrionaceae, Veillonellaceae, and Spirochaetaceae (*P* < 0.05; Figure 4B). Furthermore, at the genus level, the relative abundance of *Prevotella, unidentified F082* was increased by melatonin treatment, in contrast to that of *unidentified Succinivibrionaceae* and *Quinella* (*P* < 0.05; Figure 4C).

### Melatonin Alters Predicted Ruminal Microbial Metabolic Pathways *in vitro*

**Figure 5:**
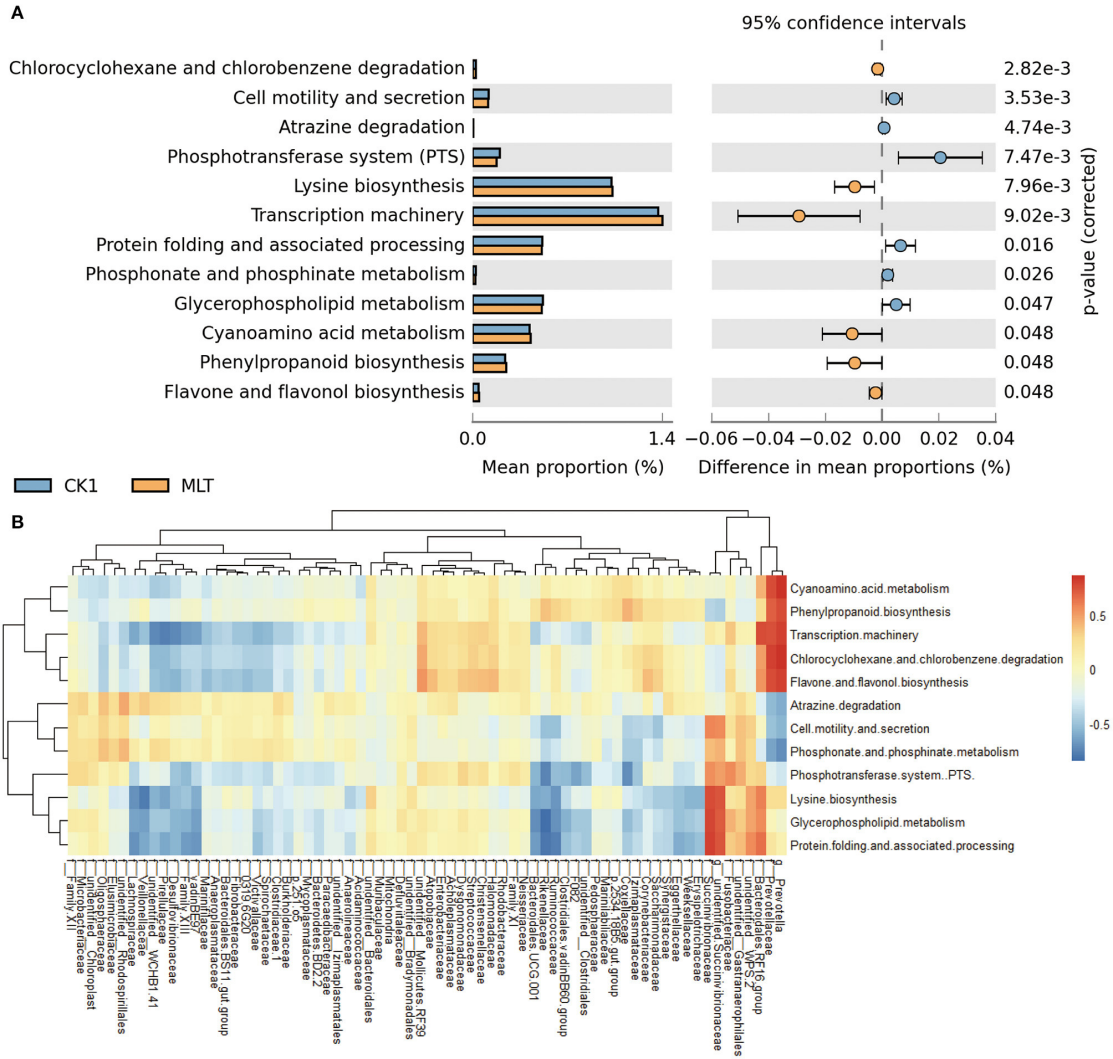
Melatonin mediated the metabolism pathway of ruminal microbiota. **(A)** Bar chart shows different metabolic pathways predicted through applying the CowPI analysis between the CK1 and MLT treatments. The blue bar and yellow bar represent CK1 and MLT, respectively. **(B)** Heatmap shows the correlation between ruminal bacteria family (contain two extra genus *Prevotella* and *unidentified Succinivibrionaceae*) and the predominant metabolic pathway affected by melatonin during *in vitro* fermentation. f for family taxa, g for genus taxa. CK0, non-rumen fluid + non-melatonin; CK1, rumen fluid + non-melatonin; MLT, rumen fluid + melatonin.

A total of 12 significantly different metabolic pathways in ruminal microbiota between the CK1 and MLT treatments were predicted by CowPI. For instance, melatonin facilitated Cell motility and secretion, Atrazine degradation, Phosphotransferase system (PTS), Protein folding and associated processing, Phosphonate and phosphinate metabolism, and Glycerphospholipid metabolism, while inhibited the Chlorcyclohexane and chlorobenzene degradation, Lysine biosynthesis, Transcription machinery, Gyanoamino acid metabolism, Phenylpropanoid biosynthesis and Flavane and flavonol biosynthesis functions of ruminal bacteria *in vitro* (Figure 5A). Additionally, family Prevotellaceae and genus *Prevotella* were negatively correlated with Atrazine degradation, Cell motility and secretion, and Phosphonate and phosphinate metabolism pathways (*P* < 0.05), but positively correlated with Chlorocyclohexane and chlorobenzene degradation, Cyanoamino acid metabolism, Flavone and flavonol biosynthesis, Phenylpropanoid biosynthesis and Transcription machinery pathways (*P* < 0.01; Figure 5B and Supplementary Figure S3). The family Succinivibrionaceae and genus *unidentified Succinivibrionaceae* had a positive correlation with Cell motility and secretion, Glycerphospholipid metabolism, Lysine biosynthesis, Phosphotransferase system (PTS), and Protein folding and associated processing of ruminal microbiota *in vitro* (*P* < 0.05; Figure 5B and Supplementary Figure S3).

## Discussion

Melatonin is an indispensable hormone regulating biological rhythms that is mainly synthesized and secreted by the pineal gland in response to environmental darkness, but limited during light conditions, which agrees with the findings that greater serum melatonin concentrations were observed at night (28). In turn, these circadian patterns regulate the rhythmicity of the biological clock in the body. At least in humans, other biological fluids such as saliva contain variable amounts of steroid hormones such as GH and PRL, and peptide hormones such as MLT partly due to diffusion from blood (29). Salivary MLT participates in the regulation of inflammatory processes, promoting antioxidant responses and cell proliferation, and contributes to the rapid healing of oral epithelial wounds (29).

The present results demonstrating that MLT in saliva of ruminants also displays circadian rhythm is similar to published work in humans and rodents. Bubenik et al. (30) were the earliest to report the presence of MLT in the ruminal muscularis and ruminal fluid of dairy cows with MLT concentrations in muscularis being greater than fluid. The origin of MLT was not evaluated. The present study not only confirmed MLT in ruminal fluid, but also that it exhibits a certain circadian rhythm, which was similar with the pattern in the serum and saliva. In addition, our detection of GH and PRL in saliva and ruminal fluid (without obvious circadian rhythms) was consistent with the findings of Koprowski et al. (31), Koyama et al. (32), and Lindell et al. (33).

Circadian oscillations of the gut microflora have been widely studied in human and mouse (34). For instance, Thaiss et al. (35) reported more than 15% of total OTUs that could be detected in mouse fecal microorganisms exhibited a circadian rhythm. This was followed by a study of Zarrinpar et al. (36), which demonstrated that 17% of total OTUs in the mouse gut microbiota exhibited a clear circadian rhythm. Unlike those previous studies, only 9% of the total OTUs in ruminal fluid demonstrated a clear and robust circadian rhythm. In fact, taking into account the fact the substantially greater number of total OTUs in rumen than intestine of human or rodents, the number of cyclical OTUs in the rumen is clearly quite considerable.

Using phyla-level assignments revealed that relative abundance of Bacteroidetes and Firmicutes oscillated diurnally in mice (36, 37). This was in accordance with the present study, as the relative abundance of Prevotellaceae, Muribaculaceae and Veillonellaceae, three dominant families of Bacteroidetes, and the Ruminococcaceae, a vital family of Firmicutes exhibited clear circadian rhythms. While the relative abundance of Proteobacteria phyla exhibited unclear rhythms in mice, the work of Liang et al. (37) inferred that the absolute abundance of Proteobacteria oscillated during the light-cycle. In contrast, the relative abundance of Proteobacteria in ruminal fluid exhibited a strong diurnal rhythm according in the present study. Succinivibrionaceae, a major family of Proteobacteria also oscillated regularly. In addition, because the phyla Bacteroidetes, Firmicutes and Proteobacteria together comprise most of the ruminal bacteria (over 97% in our study) (38), there is no doubt that nearly the entire microbiota maintains a robust circadian rhythm. Profiles of ruminal fermentation parameters, particularly the ammonia nitrogen, propionate, butyrate, and the ratio of acetate to propionate, also supported the existence of a circadian pattern.

The capacity of MLT to have a negative impact on Gram-negative bacteria is well-established (39–41). However, several recent studies have reported that MLT is not only essential to regulate the circadian rhythm of the host, but regulates the intestine by mediating metabolism of the microbiota as well as their circadian rhythms (16). Paulose and Cassone (18) demonstrated that *Enterobacter aerogenes* was sensitive to MLT and this hormone contributes to regulation of the circadian clock in *E. aneogenes*. Ma et al. (42) concluded that MLT can impact gut microbes through cytokines (e.g., IFN-γ) generated by intestinal epithelium. In addition, O'Keeffe et al. (43) demonstrated that MLT is capable to alter the metabolism of intestinal microbes in mice through NF-κB. Furthermore, Yin et al. (44) reported that administration of exogenous MLT was able to recover the disrupted diurnal rhythms of the gut microbiota in mice fed a high-fat diet.

In the present study, the families Muribaculaceae, Veillonellaceae, and Prevotellaceae, and two genera of family Succinivibrionaceae, *unidentified Succinivibrionaceae* spp. and *Quinella* spp. correlated robustly with ruminal MLT *in vivo*. Intriguingly, these bacterial taxa were dramatically influenced by supplemented MLT *in vitro*, suggesting that this hormone can mediate these cyclical microbes in the rumen. Both of the families Muribaculaceae and Prevotellaceae belong to the Bacteroidetes phylum, which demonstrated a robust circadian rhythm in response to MLT treatment in mice (44). That work also uncovered that the circadian pattern of phyla Firmicutes (contains Veillonellacea family) was impacted by MLT. Together, these findings confirm that MLT is capable of influencing circadian patterns of “rhythmic microbes” in the digestive tract including the rumen.

The fact that MLT enhanced the relative abundance of phyla Bacteroidetes and decreased Proteobacteria phyla was similar to the studies of Kim et al. (15) and Yin et al. (44) in mice. Thus, we speculate that a variety of crucial metabolic pathways within ruminal microbiota were likely to be regulated by MLT treatment. For instance, Flavonoids are a large and diverse group of secondary metabolites that are synthesized through a specific branch of the phenylpropanoid pathway (45). Flavonoids not only regulate lipid metabolism (46), but play an important role in preventing intestinal inflammation (47). Thus, the flavone biosynthesis pathway in microbiota plays a significant role on maintaining health of the host. Collectively, these results underscored the potential significance of ruminal MLT in terms of host-microbe interactions in dairy cows.

The genus *Prevotella* spp. and family Succinivibrionacae, localized to mucosal sites, have attracted much attention due to their association with a variety of systemic diseases including periodontitis, bacterial vaginosis, rheumatoid arthritis, metabolic disorders, and low-grade systemic inflammation (48). During gut dysbiosis, increased numbers of *Prevotella* were detected during persistent inflammation with HIV infections (49), metabolic syndrome (insulin-resistance) (50) as well as bowel inflammatory disease induced by NLRP6-inflammation (51). In the present study, the negative correlation between *Prevotella* numbers and atrazine concentrations suggested this microorganism could enhance atrazine accumulation. If, in fact, atrazine concentrations in the rumen are associated with changes in a major microbial species such as *Prevotella*, it could potentially lead to serious negative effects on the animal and other microbes as reported previously (52).

Beyond toxic effects, atrazine can regulate IL-1 mRNA abundance (53) and activate STAT3 signaling (54), which are well-known pro-inflammatory signals (55). Thus, we speculate that *Prevotella* could drive gut tissue inflammation through facilitating atrazine accumulation. Further studies appear warranted to study more closely the role of *Prevotella* species on physiological responses in the ruminant animal.

Recent studies provided evidence that Succinivibrionaceae species have a positive impact on milk production of lactating cows (56, 57) through their ability to utilize hydrogen and reduce production of methane (58). As such, increases in the numbers of Succinivibrionaceae should enhance availability of energy and nutrients to the host. The fact that in our study the abundance of Succinivibrionaceae was positively correlated with lysine biosynthesis suggested that this species could play a role in reducing methane. Lysine maintains the capacity to significantly inhibit both methane and carbon dioxide by shifting the equilibrium phase boundary condition to higher pressures and/or region of lower temperatures (59). Furthermore, Mu et al. (24) reported that the abundance of *Prevotella* spp. in ruminal fluid was negatively correlated with milk production. While the abundance of Succinivibrionaceae family was likely to positively associate with milk yield (25). Thus, the linkages between melatonin and these two species in this study might demonstrate that melatonin could affect milk production of dairy cows by regulating their ruminal microorganisms. Further research in this area is warranted.

## Conclusions

In summary, the present research revealed a link between melatonin and the rhythmic oscillation of the three dominant phyla Firmicutes, Proteobacteria, and Bacteroidetes *in vivo*; these diurnal cyclical bacteria were closely related with melatonin *in vivo* and were sensitive to melatonin *in vitro*, indicating melatonin is likely to play a vital role in mediating ruminal microbes and their metabolic pathway.

## Methods

### Cow Management

All experimental procedures were approved by the Animal Management Committee (in charge of animal welfare issue) of the Institute of Animal Science, Chinese Academy of Agricultural Sciences (IAS-CAAS, Beijing, China). Six lactating Holstein cows of similar weight (566.8 ± 19.6 kg), parity (3.0 ± 0.0) and milk performance (8,398.7 ± 1,392.9 kg/y) from Youran Dairy Farm (Inner Mongolia, China; Table 3). Cows were housed in individual 10-m^2^ concrete-floor pens, each of which had a separate feed bunk and watering point, feed and water was available for *ad libitum* consumption. The CALAN broadbent feeding system (American CALAN Inc., Northwood, USA) was used for feeding. Cows were trained to use the feeding system 3 weeks before the experiment to ensure that feeding behavior was normal. Cows were adapted to natural light for 2 weeks (from 12 to 25 April), fed twice a day (07:00 and 14:00), milk yield and feed intake recorded daily (Supplementary Tables S1, S2). Diet composition is reported in Table 4 (60).

**Table 3:** Body weight, number of litters, and lactation performance of lactating dairy cows.

**Cows**	**Weight**	**Lactation**	**Milk kg/y**	**Lactation days**	**Milk/d**	**Fat %**	**Protein %**	**Lactose %**
145,757	563	3	8,415	169	29.5	4.3	2.8	5.2
140,512	570	3	7,482	169	28.8	3.8	3.2	5.2
140,523	538	3	7,306	173	29.1	4.2	3.3	5.4
140,993	559	3	7,429	177	29.2	3.6	2.9	5.3
140,851	573	3	8,799	178	29.1	4.0	3.7	5.3
140,373	598	3	10,961	183	29.6	4.8	3.0	5.4
Mean	566.8	3.0	8,398.7	174.8	29.2	4.1	3.2	5.3
SD	19.6	0.0	1,392.9	5.5	0.3	0.4	0.3	0.1

**Table 4:** TMR feed formula and its nutritional level.

**Items**	**Value**
**Composition (kg DM/d)**	
Corn silage	7.36
Alfalfa hay	2.85
High moisture corn	4.20
Flaked corn	1.74
Soybean meal	1.67
Cottonseed meal	1.07
DDGS	0.90
Beer grains	0.40
Spouting corn bran	0.28
Corn gluten meal	0.46
Beet pulp	1.09
Whole cottonseed	1.50
10% Premix	1.12
Total	24.64
**Nutrient levels (g/kg DM)**	
Crude protein	160
NDF (g/kg DM)	296
Starch (g/kg DM)	246
NFC (g/kg DM)	412

### *In vitro* Culture

The *in vitro* simulated fermentation solutions were shown in Table 5. A total of three different treatments were included: (i) non-rumen fluid + non-melatonin (CK_0_); (ii) rumen fluid + non-melatonin (CK_1_); (iii) rumen fluid + melatonin (MLT). Three replicates for each treatment. The 1.2 g feed provided by Youran Dairy Farm (Inner Mongolia, China; Table 4) was weighed and used as fermentation substrates for every fermentation bottle. The standard solutions of MLT (9 ng/mL) were prepared according to Özcan and Bagci (61) using melatonin powder purchased from Sigma-Aldrich company (Sigma-Aldrich, USA). The rumen fluid was collected from four different directions of rumen of three fistula healthy Holstein cows after 2 h of morning feeding. Artificial saliva *in vitro* culture experiments were prepared according to Cone et al. (62). All incubation procedures were performed in a thermostatic water bath shaker (SHA-A, Hengfeng Instrument, China) at 39°C (63). After 24 h of cultivation, the fermentation bottle was taken out and placed in an ice water bath to stop the fermentation.

**Table 5:** Simulated ruminal fermentation solutions.

**Item**	**Treatments**		
	**CK_0_**	**CK_1_**	**MT**
Artificial saliva	119 mL	119 mL	119 mL
Rumen fluid	/	60 mL	60 mL
9 ng/mL MLT	1 mL	/	1 mL
Ultra-pure water	60 mL	1 mL	/

### Sample Collection

For the *in vivo* study, a total of 10 mL saliva, 20 mL blood, and 100 mL ruminal fluid were collected at 02:00, 10:00, and 18:00 on the first day and 06:00, 14:00, 22:00 on the second day of the experimental period following details in Negrao et al. (64), Bu et al. (65), and Wang et al. (66), respectively. For the *in vitro* study, the culture fluid samples were collected at 0, 4, 8, 12, 16, 20, and 24 h of fermentation, and immediately measure the pH value of these samples. All samples were protected from light during the collection and packaging process. For sample collection at night, a dark red light (Kodak Spa, NY, USA) with light intensity <3 lx was used to account for the fact that photosensitive intensity of cattle is 3 lx (67). All samples were packed in brown tubes (Corning Inc., NY, USA) and immediately frozen prior to storage at −80°C until analyzed. Details of ruminal fermentation parameter determination are described in our previous publication (68).

### Light Intensity, Humidity, and Temperature

The UT382 illuminance meter (UNI-T Inc., Dongguan, China) was used to measure light intensity of the barn at the sampling time. An RC-4 automatic temperature and humidity recorder (Elitech Inc., Jiangsu, China) was also used. All these records during experimental days are reported in Supplementary Table S3.

### Determination of Hormonal Concentrations

A radioimmunoassay (RIA) was used to detect the concentration of MLT, PRL, and GH in saliva, serum, and ruminal fluid. The MLT levels were measured by means of a commercial RIA kit (RE29301, IBL, Germany) that consisted of ^125^I-melatonin (69). The PRL and GH levels were tested according to the RIA methods of Schams et al. (70) and Bubenik et al. (71) using a bovine PRL antibody (AS1003.1, Immundiagnostik AG, Germany) and a bovine GH antibody (AS1006.1, Immundiagnostik AG, Germany). The intra-assay coefficients of variation (CV) for MLT, PRL, and GH were 5.4, 7.4, and 8%, and the inter-assay CV for MLT, PRL, and GH were 11.1, 14.3, and 11.4%, respectively. All samples were determined by Beijing North China Biotechnology Research Institute (Beijing, China). Ruminal fluid samples were centrifuged at 4,000 × g for 30 min at 4°C before hormonal tests.

### DNA Extraction and Sequencing

After thawing ruminal fluid, total DNA was extracted using the QIAamp DNA Stool Mini kit (Qiagen, Hilden, Germany) according to the manufacturer's protocols. The NanoDrop 1000 spectrophotometer (Thermo Fisher Scientific, MA, USA) was used to measure quality and quantity of DNA samples. The V3–V4 regions of 16S rRNA gene were amplified using the 341F/806R primer set (5′-CCTAYGGGRBGCASCAG-3′/5′-GGACTACNNGGGTATCTAAT-3′) according to Xue et al. (72). Subsequently, amplicons were purified by QIAquick gel extraction kit (Qiagen, Hilden, Germany). The Illumina Hiseq-PE 250 sequencing platform (SanDiego, USA) was used to conduct amplicon sequencing by Novogene Bioinformatics Technology Co., Ltd. (Beijing, China). The raw *in vivo* and *in vitro* amplicon sequence data generated in the study are available at the NCBI sequence read archive (SRA) under the accession number PRJNA666225 and PRJNA666235, respectively.

### Sequencing Data Analysis

The QIIME software package was used to process raw reads (73). Operational taxonomic units (OTUs) were clustered at 97% similarity cutoff (74), and the taxonomy was analyzed with the Greengenes database. JTK_CYCLE is a nonparametric algorithm to identify rhythmic components in large group size data sets (75). This experiment refers to Thaiss et al. (35), and using JTK_CYCLE source in R program to analyse circadian rhythms of bacteria. Both Bonferroni-adjusted *p*-value (ADJ.P) and Benjamini–Hochberg *q*-values (BH.Q) <0.05 were considered significant (36). CowPI analysis was used to predict functional information (PICRUSt) within the rumen microflora (76). We used the online analysis platform developed by Aberystwyth University for CowPI analysis (https://share-galaxy.ibers.aber.ac.uk). STAMP (V.2.1.3) then was used to compare the metabolic pathway predicted by CowPI and visualized the results.

### Statistical Analysis

Data analysis was carried out in Excel 2019, SAS 9.4, QIIME, GraphPad Prism software (V.8.0.2), R program (V.3.6.1), STAMP (V.2.1.3), and the Galaxy cloud platform. Hormone concentrations, relative abundance of rhythmical bacterial taxa and ruminal fermentation parameters between light/dark conditions *in vivo*, and the relative abundance of bacteria taxa *in vitro* were analyzed using paired *t*-tests, with *P* < 0.05 considered as significant. The predicted pathways were compared using the Welch's *t*-test performed by STAMP with a *P* < 0.05 being considered significant. Correlation analysis was performed using Spearman's method in R software.

## Data Availability Statement

The datasets generated for this study can be found in the NCBI sequence read archive, accession numbers PRJNA666225 and PRJNA666235.

## Ethics Statement

The animal study was reviewed and approved by Animal Management Committee (in charge of animal welfare issue) of the Institute of Animal Science, Chinese Academy of Agricultural Sciences (IAS-CAAS, Beijing, China).

## Author Contributions

JO, MW, and DB conceived and designed the study. JO, CX, and CD conducted the experiment and analyzed the animal data. JO and FL did the metataxonomic analysis. JO drafted the manuscript. AA and JL contributed to data interpretation and writing of the manuscript. JO, MW, DB, and LM contributed to the writing of the manuscript. JL edited the final version of the manuscript. All the authors revised and edited the manuscript.

## Funding

This work was supported by the Open subject of State Key Laboratory of Animal Nutrition (Grant 2004DA125184F1715), Key program of State Key Laboratory of Sheep Genetic Improvement and Healthy Production (2021ZD07 and 2021ZD01), Science and Technology Innovation Project of Chinese Academy of Agricultural Sciences (Grants ASTIP-IAS07 and CAAS-XTCX2016011-01), and Jiangsu Province Graduate Research and Innovation Project (Grant XKYCX19_121).

## Conflict of Interest

The authors declare that the research was conducted in the absence of any commercial or financial relationships that could be construed as a potential conflict of interest.

## Publisher's Note

All claims expressed in this article are solely those of the authors and do not necessarily represent those of their affiliated organizations, or those of the publisher, the editors and the reviewers. Any product that may be evaluated in this article, or claim that may be made by its manufacturer, is not guaranteed or endorsed by the publisher.

## Abbreviations

MLT: Melatonin
GH: Growth hormone
PRL: Prolactin
BH.Q: Benjamini-Hochberg *q*-value
ADJ.P: Bonferroni-adjusted *p*-value
OTUs: Operational taxonomic units
NH_3_-N: Ammonia nitrogen
A:P: Ratio of acetate to propionate
SRA: Sequence read archive
RIA: Radioimmunoassay.
