# Supplementary material for: Ruminal Microbes Exhibit a Robust Circadian Rhythm and Are Sensitive to Melatonin
Source: Front Nutr. 2021 Oct 25;8:760578. doi: 10.3389/fnut.2021.760578 (PMC8573100; doi:10.3389/fnut.2021.760578)
Supplement: Supplementary file 1 [file Data_Sheet_1.PDF]

Supplementary Material

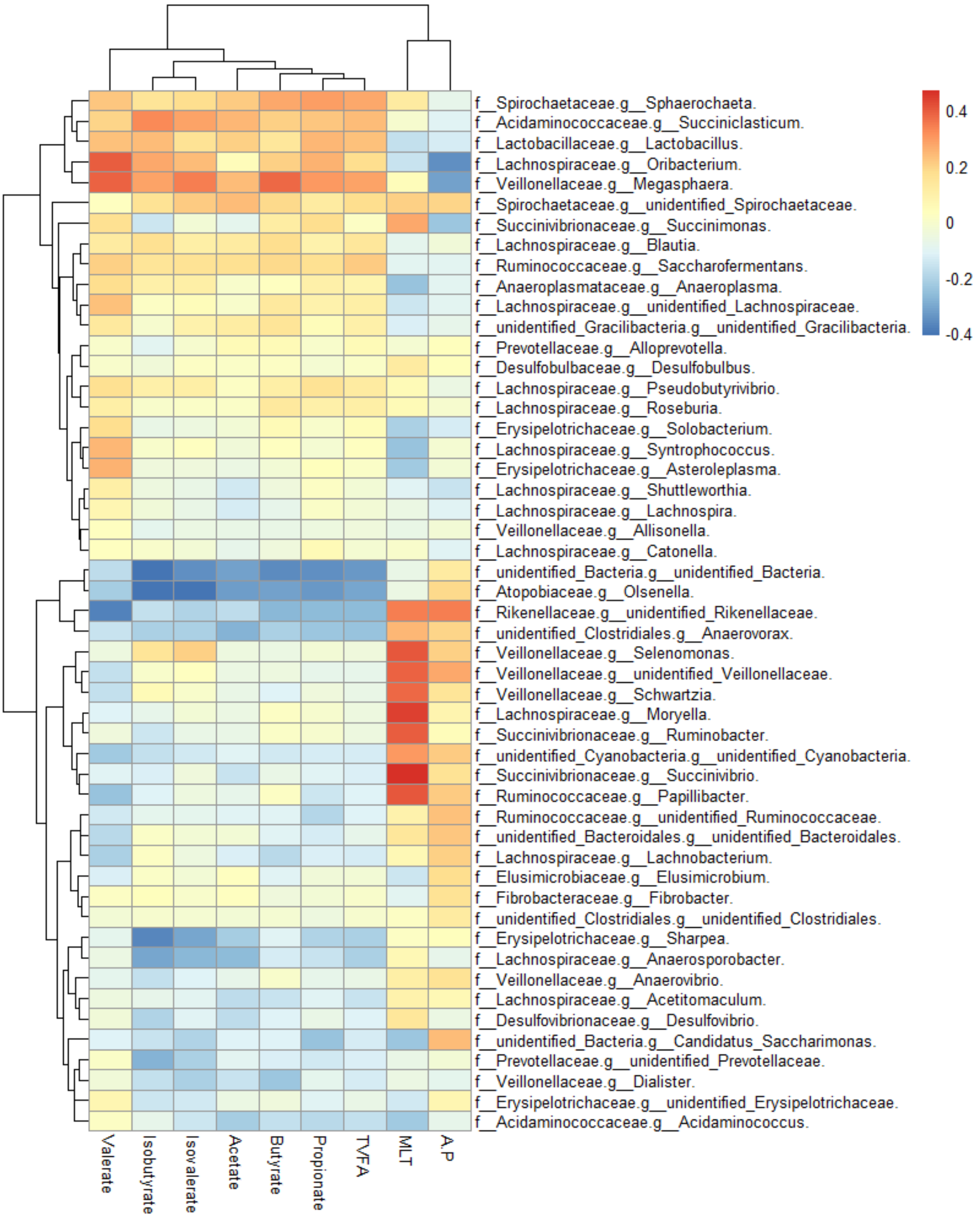

Supplementary Figure S1. The ruminal microbes correlated with ruminal melatonin and fermentation parameters. Cluster heatmap figure shows the correlation between the bacterial genus of rumen and several ruminal metabolites compassing melatonin and volatile fatty acids. MLT, melatonin; TVFA, total volatile fatty acids; A:P, the ratio of acetate to propionate; f, family; g, genus.

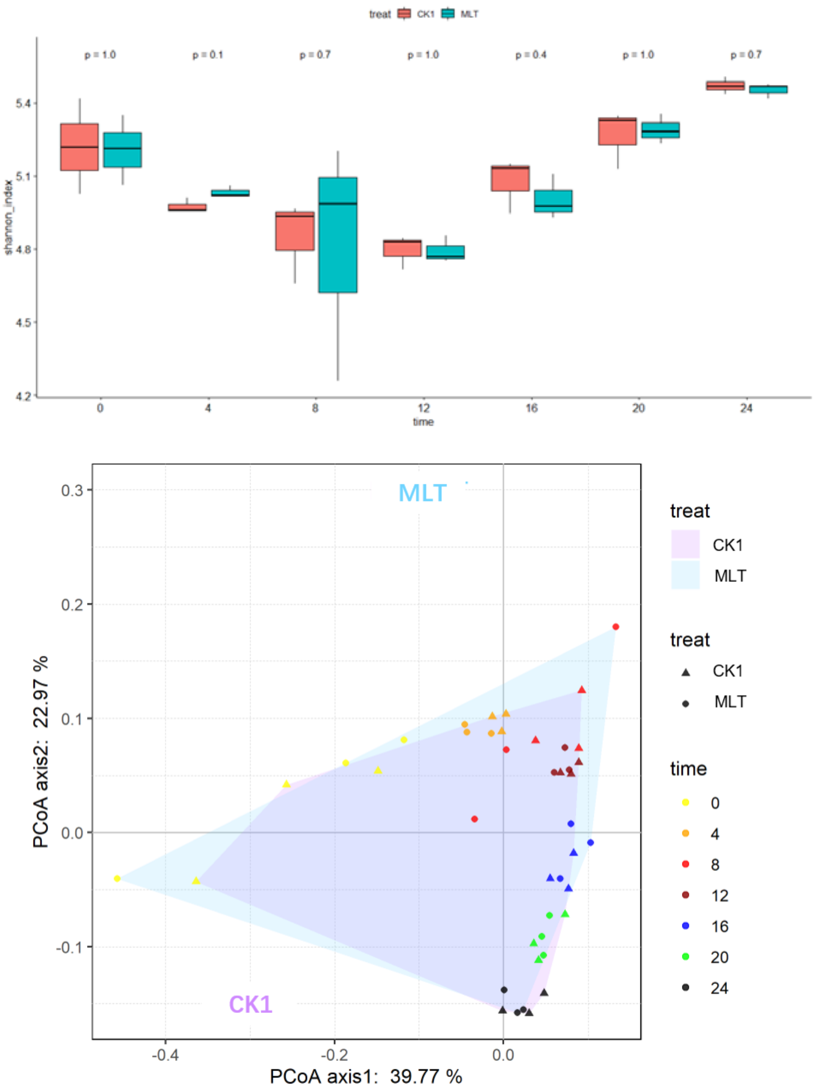

Supplementary Figure S2. Shannon index and beta diversity in treatment of CK1 and MLT at different time of fermentation *in vitro*.

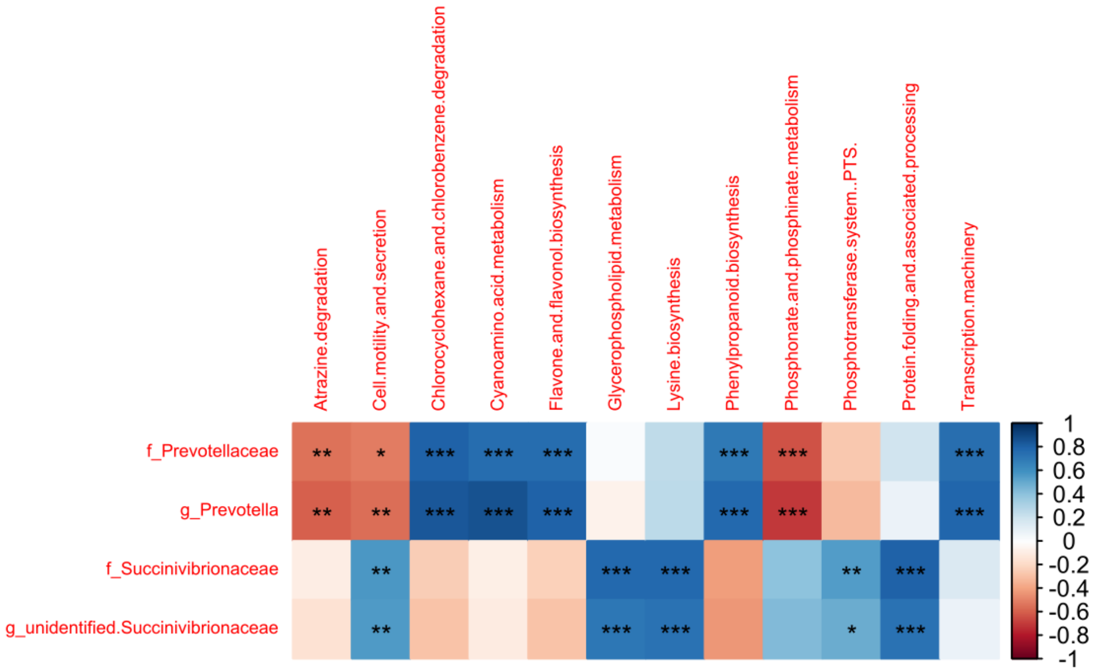

Supplementary Figure S3. Correlation analysis between ruminal microbes and differential predicted metabolic pathways. f, family; g, genus. * P<0.05, **P<0.01, ***P<0.001.

Supplementary Table S1. Daily milk yield of experimental lactating cows / kg

Items	Cows					
	145757	140512	140523	140993	140851	140373
Adaptation						
1 d	30.9	30.1	30.5	30.1	31.2	30.8
2 d	30.1	29.5	29.0	28.5	28.7	29.8
3 d	30.4	28.4	29.2	30.0	29.9	29.8
4 d	30.3	30.1	28.0	28.7	27.7	30.3
5 d	28.5	29.8	28.1	29.7	29.2	29.2
6 d	29.5	29.5	28.9	29.6	27.2	28.3
7 d	30.5	27.2	28.7	29.2	27.3	30.6
8 d	27.7	29.0	30.5	28.7	27.5	30.1
9 d	31.0	26.8	28.1	29.4	30.5	29.8
10 d	30.7	28.1	30.0	28.5	29.2	28.5
11 d	30.3	27.0	28.0	29.0	28.1	29.4
12 d	28.7	29.2	29.2	30.2	29.5	30.4
13 d	29.9	29.5	29.3	29.7	30.9	30.4
14 d	28.2	29.8	29.5	28.8	30.4	28.6
Experimental						
1 d	27.5	28.8	28.7	29.4	30.9	29.3
2 d	28.4	27.7	29.8	28.4	28.1	28.2
Mean	29.5	28.8	29.1	29.2	29.1	29.6
SEM	0.106					
P-value	0.052					

Supplementary Table S2. Feed intake of experimental lactating cows / kg

Items	Cows					
	145757	140512	140523	140993	140851	140373
Adaptation						
1 d	21.63	19.47	24.75	23.43	18.76	25.51
2 d	16.70	22.15	21.93	19.78	20.40	27.92
3 d	17.68	22.43	24.77	27.10	23.80	25.92
4 d	15.72	18.62	25.17	24.88	21.31	24.73
5 d	15.35	21.84	23.94	27.20	25.02	28.20
6 d	16.21	19.92	26.58	26.60	25.98	26.76
7 d	27.29	23.10	27.12	34.11	29.18	30.69
8 d	19.79	23.97	28.15	26.70	24.66	30.41
9 d	20.84	25.02	29.20	27.75	20.36	27.26
10 d	17.32	26.53	28.40	28.42	27.76	28.59
11 d	28.59	24.29	27.92	26.17	28.25	32.11
12 d	30.33	25.05	28.32	25.64	25.04	28.93
13 d	28.94	23.97	26.47	28.95	30.97	32.82
14 d	27.29	25.14	26.89	26.58	22.77	26.43
Experimental						
1 d	21.65	23.33	25.58	29.06	26.74	30.13
2 d	22.57	26.53	28.40	28.42	27.76	24.59
Mean	21.74	23.21	26.47	26.92	24.92	28.19
SEM	0.397					
P-value	0.167					

Supplementary Table S3. Changes in light intensity, temperature and humidity

Items	Time					
	02:00	06:00	10:00	14:00	18:00	22:00
Light intensity/lx	0.5	493.9	1670.7	5176.3	737.4	0.9
Temperature/℃	9.2	6.4	15.9	21.4	20.7	13.7
Humidity/%	39.8	47.3	28.6	21.4	20.2	39.5
